# Quantitative Comparison of Bone and Dental Morphology Between Invisalign^®^ Integrated CBCT Images and Conventional CBCT Images

**DOI:** 10.3390/dj14090580

**Published:** 2026-09-09

**Authors:** Hind W. Albilali, Abdulaziz S. Alamri, Suliman Y. Shahin, Naif N. Almasoud, Osama A. Alsulaiman, Dalal M. Almazrou, Ahmed A. Alsulaiman

**Affiliations:** 1 Fellowship in Orthodontics, Department of Preventive Dental Sciences, College of Dentistry, Imam Abdulrahman Bin Faisal University, P.O. Box 1982, Dammam 31441, Saudi Arabia; 2 Department of Preventive Dental Sciences, College of Dentistry, Imam Abdulrahman Bin Faisal University, P.O. Box 1982, Dammam 31441, Saudi Arabia; absalamri@iau.edu.sa (A.S.A.); sshahin@iau.edu.sa (S.Y.S.); nnalmasoud@iau.edu.sa (N.N.A.); oaalsulaiman@iau.edu.sa (O.A.A.); dmalmazrou@iau.edu.sa (D.M.A.)

**Keywords:** cone-beam computed tomography, orthodontics, alveolar bone, reproducibility of results

## Abstract

**Background/Objectives:** A recent feature in the Invisalign^®^ system allowed integration of cone-beam computed tomography (CBCT) with intraoral scans, thereby enabling visualization of the tooth roots and surrounding alveolar bone. Here, we aimed to compare differences between anatomical measurements obtained from integrated CBCT using Invisalign^®^ software and conventional CBCT images. **Methods:** In this cross-sectional study, we included 45 adult patients (mean age 29.53 ± 7.66 years). Alveolar bone width was measured at the crestal, mid-root, and apical levels and the root length, maxillary and mandibular outer and inner transverse widths, and symphyseal width were assessed. Intra-examiner reliability was assessed using intraclass correlation coefficients. Paired t-tests were used to compare the two methods, and Bland–Altman analysis was used to evaluate agreement. **Results:** Intra-examiner reliability demonstrated excellent agreement for all measurements (intraclass correlation coefficients > 0.90). Integrated CBCT models showed greater alveolar bone width, symphyseal width, and interbuccal transverse measurements than conventional CBCT, whereas the root length and interlingual transverse widths were underestimated. Most of the differences between the two methods were statistically significant (*p* < 0.05), although 5 of the 12 crestal measurements did not reach statistical significance (*p* > 0.05). For most measurements, the mean absolute differences were below 0.7 mm, except for root length, which showed larger discrepancies. Bland–Altman analysis revealed good agreement between the methods, with most observations lying within the 95% limits of agreement and no evident proportional bias. **Conclusions:** Most measured variables showed significant differences between integrated and conventional CBCT (*p* < 0.05), ranging from 0.7 mm to 1.3 mm; however, at the crestal level, 5/12 measurements showed no statistically significant difference between methods (*p* > 0.05).

## 1. Introduction

Esthetics have consistently been identified as a primary concern in patients undergoing orthodontic treatment using fixed appliances [1,2]. In 1999, Align Technology (Santa Clara, CA, USA) introduced a new treatment modality, Invisalign^®^ clear aligner therapy [2]. In addition to improved esthetics, clear aligners are removable, which allows patients to perform oral hygiene procedures more easily than when using fixed appliances, where plaque control is more challenging [2]. Clear-aligner therapy is based on computer-aided design and manufacturing technologies. Tooth movement is digitally planned, and a series of custom-made aligners are fabricated to achieve the planned movements sequentially [2,3]. Digital orthodontic records, including digital radiographs and digital study models, have become increasingly common in clinical practice and are often combined with software programs that aid clinicians in diagnosis and treatment planning [4].

Advances in three-dimensional imaging have further influenced orthodontic diagnosis. Cone-beam computed tomography (CBCT) has been used in dentistry since 1998 and provides three-dimensional visualization of craniofacial structures [5,6]. Unlike conventional two-dimensional radiographs, CBCT allows the visualization of anatomical structures without superimposition or distortion and enables assessment in all three spatial planes [5]. In addition, large-field-of-view CBCT scans can be used to generate reconstructed panoramic and cephalometric images from the same dataset, provided that the required anatomical regions are included [7]. CBCT is particularly useful for evaluating alveolar bone morphology. Orthodontic tooth movement may result in alveolar bone dehiscence or fenestration, especially when tooth movement exceeds the anatomical boundaries of the alveolar housing. Therefore, assessment of alveolar bone height and thickness before treatment is important. CBCT is currently considered the most reliable imaging method for this purpose [8,9]. In September 2022, Align Technology introduced a CBCT integration feature into Invisalign^®^’s treatment planning software. This feature allows CBCT data to be combined with intraoral scans, enabling visualization of the tooth roots and surrounding alveolar bone within the Invisalign^®^ software, which provides additional anatomical information that may assist clinicians during treatment planning [10].

Although many studies have examined bone and root morphology using conventional CBCT, the integration of CBCT with intraoral scans within the Invisalign^®^ system is a relatively new feature. To our knowledge, this study is the first to quantitatively evaluate its measurement accuracy and agreement with conventional CBCT. Therefore, in this study, we aimed to compare the differences between anatomical measurements obtained from integrated CBCT using the Invisalign^®^ software and conventional CBCT images.

## 2. Materials and Methods

### 2.1. Study Setting and Design

This cross-sectional retrospective study was conducted at a private orthodontic practice. The data were collected between September 2024 and September 2025.

### 2.2. Study Population

The records of existing patients who underwent Invisalign^®^ treatment with integrated CBCT and were treated by a single orthodontic consultant were included in this study. Patients were excluded if they had no available CBCT records; presented with systemic or metabolic diseases, periodontal conditions, periapical lesions, syndromes, or maxillofacial deformities; or had partial or low-resolution CBCT images.

### 2.3. Variables

**Figure 1:**
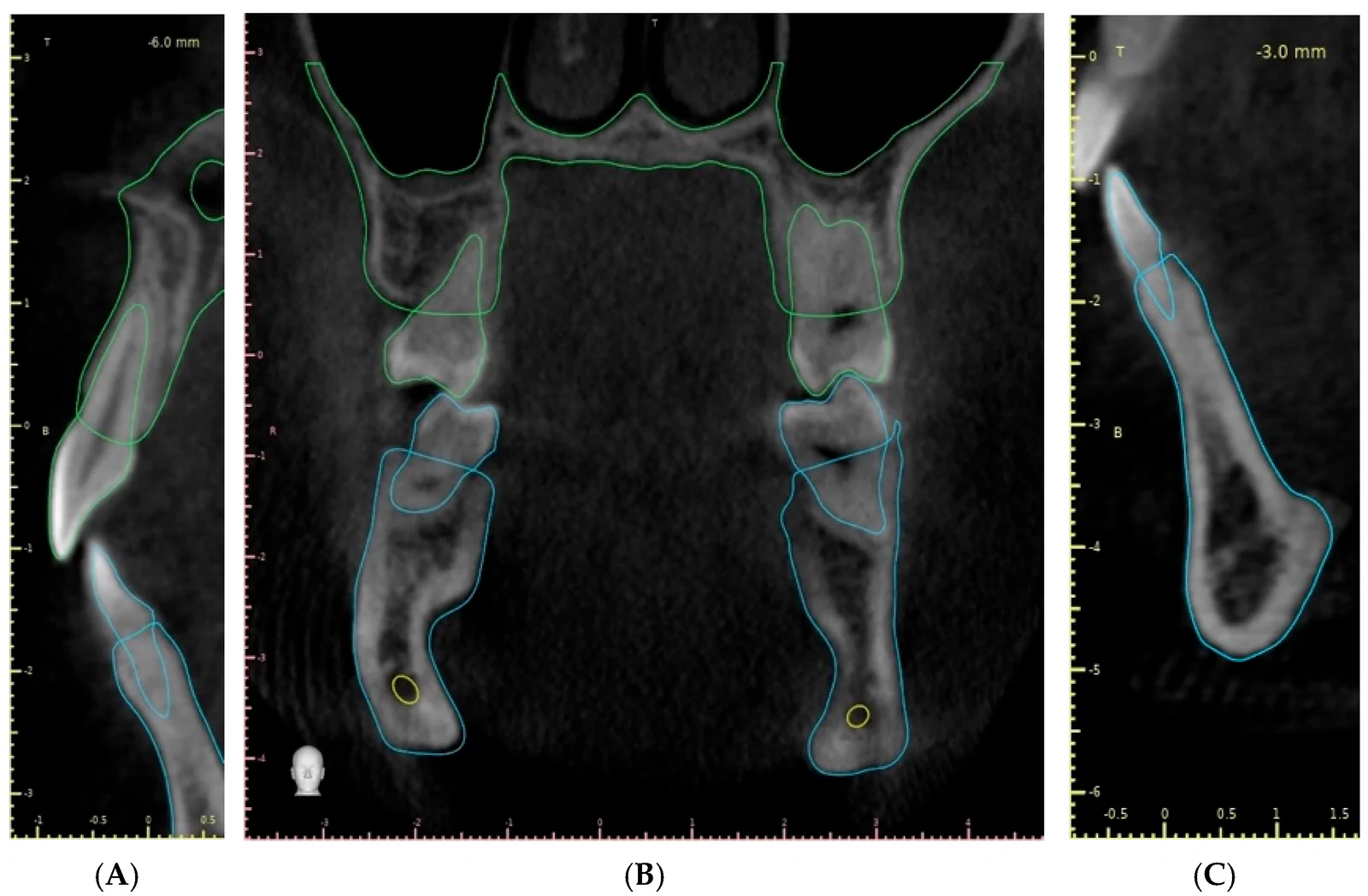
Study variables and CBCT measurement planes. (**A**) Sagittal cross-sectional view illustrating the assessment of alveolar bone width and crown-root length along the long axis of the tooth. (**B**) Coronal cross-sectional view demonstrating the evaluation of maxillary and mandibular transverse widths, including both outer and inner dimensions. (**C**) Sagittal cross-sectional view showing the measurement of symphyseal width in the mandibular region. CBCT, cone-beam computed tomography.

**Figure 2:**
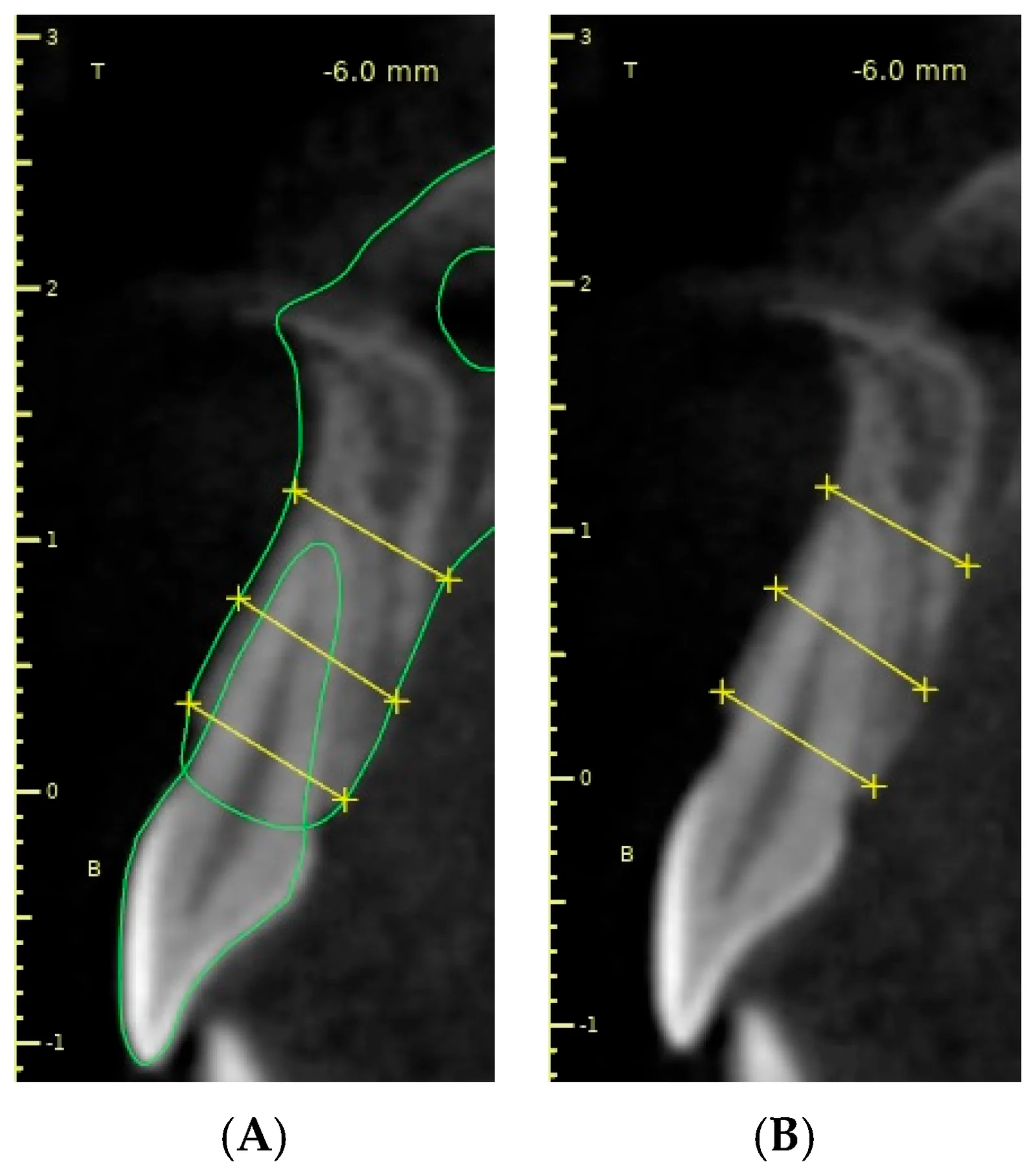
Linear measurement protocol on integrated and conventional CBCT images. (**A**) Cross-sectional view obtained from the integrated CBCT model, illustrating linear measurements of alveolar bone width performed at predefined vertical levels along the tooth root. (**B**) Corresponding cross-sectional view obtained from the conventional CBCT image at the same slice position and orientation, showing the repeated linear measurements. CBCT, cone-beam computed tomography.

For each participant, linear measurements were obtained using both integrated CBCT combined with intraoral scans and conventional CBCT. For each participant, 61 linear measurements, including 48 measurements from the alveolar bone region, 1 measurement of the mandibular symphysis width, and 12 measurements of the maxillary and mandibular transverse widths, were obtained using each method, resulting in 122 paired measurements in total. A Microsoft Excel (Version 16.112.3) spreadsheet included patient demographic data (age and sex), group assignment, and differences between the two groups in horizontal bone width, crown–root length, maxillary and mandibular widths, and symphysis width. The study variables and CBCT measurement planes are shown in Figure 1. Linear measurements were first obtained from the integrated CBCT models and were subsequently repeated on the corresponding conventional CBCT images to calculate the measurement differences, as illustrated in Figure 2. All the measurements were performed by a single examiner. The variables included in this study were selected based on previously published methodologies [11,12,13]. The definitions and measurement details are summarized in Table 1. Measurements were performed bilaterally at the central incisors, first premolars, and first molars in both arches for sagittal bone width and crown–root length. Transverse maxillary and mandibular widths were assessed at the first-molar region, and a single sagittal measurement was obtained at the mandibular symphysis.

### 2.4. Data Sources and Measurements

Conventional CBCT data were exported from the Carestream 3D (CS 9300 Premium; Carestream Dental, Atlanta, GA, USA) imaging system (85 kV, 4 mA, 10.48-s exposure, voxel size 300 µm × 300 µm × 300 µm, full field of view) as Digital Imaging and Communications in Medicine (DICOM) files. CBCT data integrated with intraoral scans were exported from the Invisalign^®^ ClinCheck platform (Version 6.0) as standard tessellation language (STL) files.

### 2.5. STL–DICOM Superimposition

**Figure 3:**
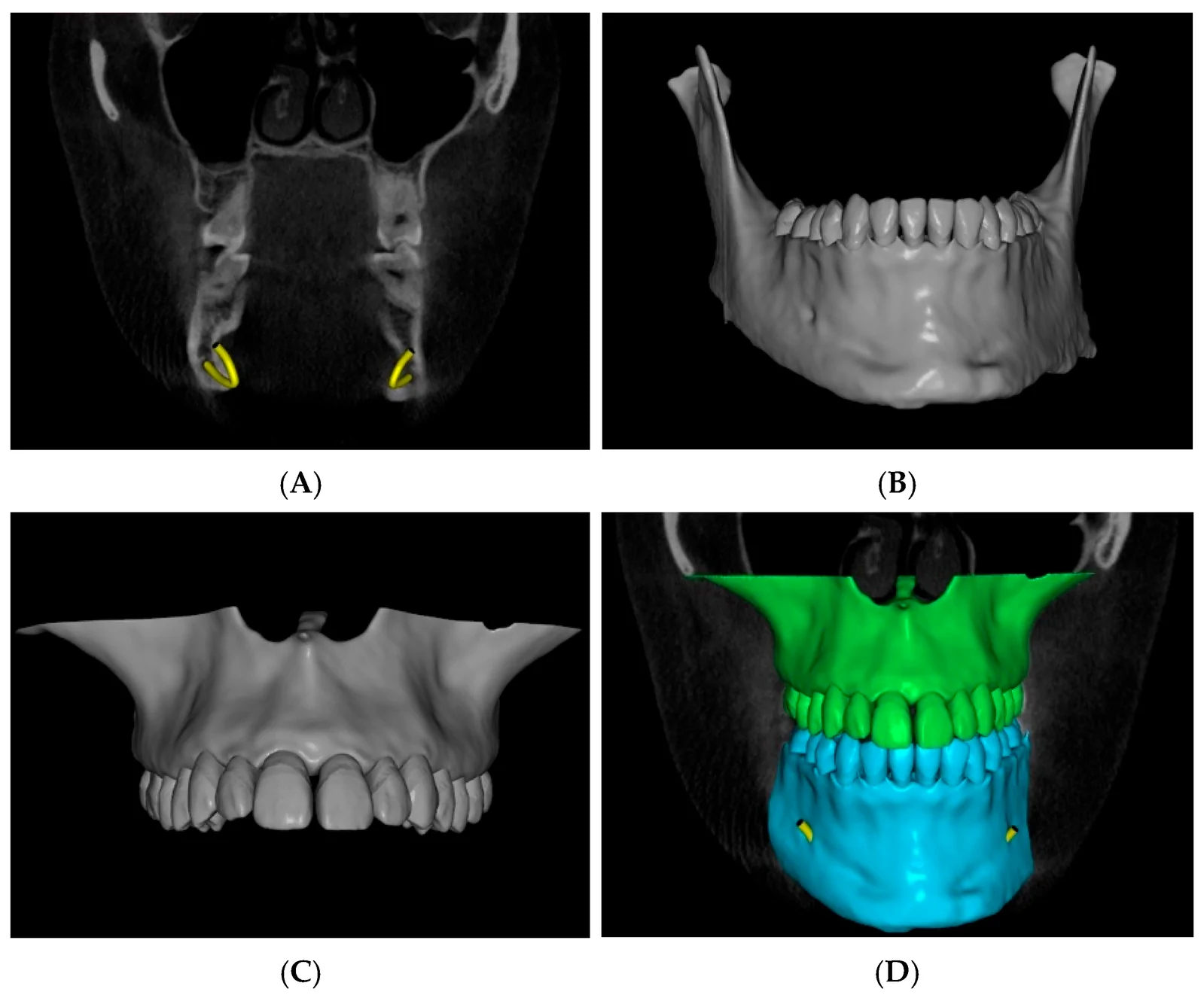
Workflow of integrated CBCT model generation and superimposition. (**A**) Conventional CBCT image. (**B**,**C**) Three-dimensional dentoalveolar models exported from Invisalign^®^ ClinCheck Version 6.0. (**D**) Superimposition of the integrated CBCT models onto conventional CBCT data. CBCT, cone-beam computed tomography.

Superimposition was performed using Blue Sky Plan 4 software (v 4.9.4.2; Blue Sky Bio, LLC, Libertyville, IL, USA). Both STL and DICOM files were imported, and automatic registration was achieved by matching six corresponding dental hard-tissue landmarks in each arch: the mid-incisal edges of the right and left central incisors, the cusp tips of the right and left canines, and the mesiobuccal cusp tips of the right and left first molars. These points were used to obtain the initial three-dimensional alignment. After the initial registration, surface-based automated registration of the CBCT and intraoral scan data was refined using buccal and lingual surfaces as the optimal overlap areas. To ensure that measurements were obtained at the same anatomical location in both datasets, measurements were first performed on the integrated CBCT model. The integrated CBCT overlay was then hidden while maintaining the same slice position and orientation, allowing the corresponding measurements to be repeated using the conventional CBCT image. The workflow for integrated CBCT model generation and STL–DICOM superimposition is illustrated in Figure 3. This methodology was previously described by Couso-Queiruga et al. [14,15], who used a similar approach for gingival thickness assessment.

### 2.6. Sample Size

The minimum sample size was calculated using G*Power software (version 3). The calculation was based on a two-sided paired t-test to detect differences between two related measurements, with a significance level of α = 0.05, statistical power of 0.80, and a medium standardized paired effect size (Cohen’s dz = 0.43). A minimum sample size of 45 participants was required.

### 2.7. Quantitative Variables and Statistical Methods

All data were anonymized before analysis and processed using SAS software (version 9.4; SAS Institute Inc., Cary, NC, USA). To assess intra-examiner reliability, measurements from ten randomly selected patients were repeated after a 2-week interval, and reliability was quantified using the intraclass correlation coefficient. Descriptive analyses including means and standard deviations were also performed. The paired t-test was used to evaluate the statistical significance of differences between measurements obtained from the integrated CBCT models and conventional CBCT images. Agreement between the two measurement methods was evaluated using Bland–Altman analysis, with calculation of the mean difference (bias) and corresponding 95% limits of agreement to evaluate systematic variation and agreement range. Bland–Altman plots were produced using BA-plotteR, a web-based tool developed for generating Bland–Altman plots and limits of agreement. This platform allows for standardized, reproducible visualization of the agreement between quantitative measurement methods and facilitates the identification of potential systematic bias across the measurement range [16].

### 2.8. Ethical Considerations

The study protocol received approval from the Institutional Review Board at Imam Abdulrahman Bin Faisal University (IRB-PGS-2024-02-375), as stated in the Institutional Review Board Statement. Since the study was retrospective and based on existing clinical records, the requirement for individual informed consent was waived by the IRB. Before the analysis, patient identifiers were removed and each record was given a study code. The Invisalign^®^ and CBCT datasets exported for the study contained no patient names or other direct identifying information.

## 3. Results

### 3.1. Intra-Examiner Reliability

Measurements from ten randomly selected patients were repeated after a 2-week interval. The intra-examiner reliability was excellent across all variables, with intraclass correlation coefficient values exceeding 0.90.

### 3.2. Participant Characteristics

A total of 45 participants were included in this study. Of these, eight were men (17.78%) and 37 were women (82.22%). The mean age of the sample was 29.53 years (standard deviation = 7.66).

### 3.3. Comparison of Bone and Dental Morphology Obtained from Integrated CBCT Models and Conventional CBCT Images

The comparisons between the integrated CBCT and conventional CBCT measurements for alveolar bone width, transverse dimensions, and symphyseal width are presented in Table 2, with significant differences observed across several measurements.

#### 3.3.1. Sagittal Bone Width Across Different Vertical Levels

Comparison between integrated CBCT and conventional CBCT revealed a clear level-dependent discrepancy in sagittal bone width. At the crestal level, sagittal bone width values obtained from integrated CBCT were slightly larger than those from conventional CBCT. Statistically significant differences were found in 7 of the 12 variables (58%), with a mean absolute difference of 0.155 mm. At the mid-root level, the mean absolute difference was 0.296 mm. All the variables at this level (12/12) showed statistically significant differences. At the apical level, all variables showed statistically significant differences. The mean absolute difference further increased to 0.417 mm. The sagittal bone width values obtained using integrated CBCT were consistently larger than those obtained by conventional CBCT, with the greatest differences observed at the apical level.

#### 3.3.2. Root Length

The root length demonstrated the greatest discrepancy among all parameters. The mean difference was −1.258 mm, indicating that conventional CBCT measured longer root lengths than integrated CBCT. All root length variables showed statistically significant differences between methods.

#### 3.3.3. Transverse Maxillary and Mandibular Skeletal Width

The outer cortical width measurements were larger on integrated CBCT than on conventional CBCT. This pattern was observed across all the outer cortical variables. The mean difference was 0.502 mm, and all variables showed statistically significant differences. For the inner cortical width, the measurements obtained using integrated CBCT were smaller than those obtained using conventional CBCT. Mean differences were negative, with an average value of −0.359 mm. Differences in most inner cortical variables were statistically significant, except for MDI3.

#### 3.3.4. Symphysis Measurements

A small but statistically significant difference was observed in symphyseal width. The measurements obtained from integrated CBCT were slightly larger than those obtained from conventional CBCT, with a mean difference of 0.131 mm.

#### 3.3.5. Bland–Altman Analysis

Bland–Altman plots were used to assess the agreement between integrated CBCT and conventional CBCT. The agreement varied according to the measurement level and variables. A small positive mean bias was observed at the crestal level. Most data points were close to the mean difference. These values were within the 95% limits of agreement. A positive mean bias was observed at the mid-root and apical levels, and the integrated CBCT measurements were slightly larger at these levels, with statistically significant differences. Most of the observations were within the 95% limits of agreement. For root length, a negative mean bias was observed for all teeth. The mean differences were below zero. The root length values obtained from integrated CBCT were smaller than those obtained using conventional CBCT. The limits of agreement were wider than those for the alveolar bone width. For transverse skeletal measurements, similar patterns were noted at all evaluated vertical levels (2, 6, and 10 mm); the outer (buccal) transverse widths in both the maxilla and mandible showed a small positive bias, whereas the inner (palatal/lingual) transverse widths showed a negative bias. Most measurements remained within the 95% limits of agreement. For symphyseal width, the Bland–Altman plot showed a small positive bias with a symmetrical distribution of data points. All observations were within the 95% limits of agreement. Bland–Altman plots for individual teeth and measurement levels are provided in the Supplementary Figures. Numerically, the mean bias ranged from −0.139 to 0.433 mm at the crestal level (95% limits of agreement across individual measurements: approximately −0.84 to 1.55 mm), from 0.086 to 0.464 mm at the mid-root level (approximately −0.67 to 1.38 mm), and from 0.208 to 0.612 mm at the apical level (approximately −0.98 to 1.84 mm). Root-length bias ranged from −1.678 to −1.011 mm, with the widest limits extending from approximately −3.62 to 0.67 mm. For transverse and symphyseal measurements, bias ranged from −0.601 to 0.641 mm; the corresponding numerical values for each variable are provided in Supplementary Table S1.

## 4. Discussion

Earlier digital orthodontic systems relied solely on surface-based intraoral scans, limiting virtual tooth movement to the crowns. The integration of CBCT with intraoral scans has introduced more anatomically complete modeling that visualizes crowns, roots, and their surrounding alveolar housing, transforming digital setups into biologically informed diagnostic tools that relate planned tooth movements to skeletal anatomy. Therefore, validating the accuracy of these integrated CBCT models is clinically important, as these models allow clinicians to evaluate bone and tooth anatomy directly within an integrated dataset without repeatedly reviewing conventional CBCT data. Recent studies have also emphasized the clinical value of combining crown, root, and alveolar bone information in digital orthodontic planning and have shown that CBCT–intraoral scan integration can provide accurate three-dimensional root models [17].

CBCT was used as the reference standard. Li et al. reported small and insignificant mean differences between CBCT measurements and the gold-standard reference measurements for alveolar bone height and thickness in a systematic review and meta-analysis [9]. Couso-Queiruga et al. [14,15] validated the digital superimposition of CBCT and intraoral scans for soft tissue evaluation, demonstrating no significant differences between digital and clinical measurements, confirming the high accuracy of this method. While these studies established the reliability of digital superimposition for soft tissue assessment, the present study extended this approach to bone and dental morphology.

In the assessment of sagittal alveolar bone width, the findings revealed clear level-dependent discrepancies between integrated CBCT and conventional CBCT. The integrated model slightly overestimated the alveolar bone width near the crest, with 7 of the 12 variables showing statistical significance, indicating relatively good agreement at this level. In contrast, the mid-root and apical regions exhibited progressively greater overestimation. The root length demonstrated an opposite pattern, with the integrated model consistently underestimating the root length relative to conventional CBCT across all teeth. In the transverse dimension, integrated CBCT overestimated the outer (buccal) cortical width and underestimated the inner (palatal/lingual) cortical width of both the maxilla and mandible. The observed discrepancies were slightly greater in the maxilla (0.38–0.64 mm) than in the mandible (0.02–0.63 mm), which may be related to differences in cortical bone anatomy. In the symphyseal region, the integrated CBCT showed a small overestimation of the width, with a mean difference of 0.131 mm. In general, the differences between integrated CBCT and conventional CBCT measurements reached statistical significance for most variables. This should be considered when interpreting the measurements obtained from integrated CBCT models.

The smallest discrepancy in the transverse width measurements was observed in the mandible at the apical level, specifically 10 mm apical to the cemento-enamel junction of the mandibular first molar. Wood et al. reported that measurements obtained in this region demonstrated high agreement with the physical reference values, with most discrepancies remaining within 1 mm and occurring infrequently [18]. A similar pattern was observed in the present study, where integrated CBCT differed from conventional CBCT by only −0.018 mm in interlingual width and 0.33 mm in interbuccal width. The higher accuracy of mandibular measurements compared with maxillary measurements is consistent with previous studies, which consistently reported that the mandibular alveolar and cortical bones are significantly thicker and denser than the maxillary bones [8,18,19,20]. In addition, the posterior alveolar regions were shown to have greater bone thickness than the anterior regions, a finding reported by Wood et al., which further supports the improved measurement reliability at posterior mandibular sites [18]. Evangelista et al. and Porto et al. described a progressive increase in alveolar and cortical bone thicknesses toward the apical region [8,19]. Similarly, Wood et al. associated improved CBCT measurement accuracy at the apical level with increased cortical bone thickness [18]. Cassetta et al. further demonstrated that both cortical thickness and density increased from the alveolar crest toward the basal bone, particularly in the mandible [20]. Conversely, clinically inaccurate measurements have been reported more frequently in maxillary regions, particularly in the molar area, where thin buccal cortical plates may compromise measurement accuracy, as highlighted by Wood et al. [18].

In this study, only linear measurements were performed. Therefore, future studies should include volumetric assessments and three-dimensional surface deviation analyses to better evaluate integration accuracy. In addition, the analysis was limited to the Invisalign^®^ integration platform. Future studies should include other clear aligner systems, including Spark (Ormco Corporation, Brea, CA, USA) and SureSmile (Dentsply Sirona, Charlotte, NC, USA), to clarify whether similar results occur across different software platforms. Accuracy may also be enhanced through advances in segmentation and image alignment. Because 61 paired comparisons were examined, no formal multiplicity correction was applied. The resulting *p* values should therefore be interpreted as supportive rather than confirmatory, since the primary objective was to evaluate agreement between measurement methods using Bland–Altman analysis.

## 5. Conclusions

Across all measured variables, including the alveolar bone width, symphysis width, maxillary and mandibular transverse widths, and root length, the absolute mean differences remained below 0.7 mm, except for root length, which demonstrated a slightly larger discrepancy of approximately 1.3 mm. Integrated CBCT can be used to assess alveolar and symphyseal bone morphology and evaluate potential dehiscence and fenestration near the alveolar crest. However, in coronal views, clinicians should interpret buccal bone contours with caution, as the integrated model tends to slightly overestimate the buccal bone thickness. Overall, the integrated CBCT model consistently overestimated the alveolar bone width, symphysis width, and interbuccal maxillary and mandibular widths, whereas it underestimated the root length and interlingual maxillary and mandibular widths.

## Figures and Tables

**Table 1:** Summary of study variables, measurement definitions, and landmarks.

Variable	Definition/Measurement	Levels/Points Measured	Software/View Used
Sagittal bone width	Measured as described by Cho HJ et al. (2019) [11]. Linear distance between buccal and lingual/palatal cortical plates at standardized root levels.	D1: Alveolar bone width measured at the crestal level D2: Alveolar bone width measured at the mid-root level D3: Alveolar bone width measured at the apical level	Blue Sky Plan—Sagittal sections
Crown–root length	The distance measured along the long axis of the tooth from the incisal or occlusal reference point to the root apex.	Central incisors: incisal edge → apex First premolars: Upper: palatal cusp → palatal apex (or both apices) Lower: cusp tip → apex First molars: Upper: palatal cusp → palatal root apex Lower: distal cusp → distal root apex	Blue Sky Plan—Sagittal sections
Maxillary width—outer	Measured according to Yi L et al. (2021) [12]. Buccal cortical plate to buccal cortical plate distance.	Mx1: 2 mm apical to U6 CEJ Mx2: 6 mm apical to U6 CEJ Mx3: 10 mm apical to U6 CEJ	Blue Sky Plan—Coronal sections
Maxillary width—inner	Measured according to Yi L et al. (2021) [12]. Palatal cortical plate to palatal cortical plate distance.	Mx1: 2 mm apical to U6 CEJ Mx2: 6 mm apical to U6 CEJ Mx3: 10 mm apical to U6 CEJ	Blue Sky Plan—Coronal sections
Mandibular width—outer	Measured according to Yi L et al. (2021) [12]. Buccal cortical plate to buccal cortical plate distance.	Md1: 2 mm apical to L6 CEJ Md2: 6 mm apical to L6 CEJ Md3: 10 mm apical to L6 CEJ	Blue Sky Plan—Coronal sections
Mandibular width—inner	Measured according to Yi L et al. (2021) [12]. Lingual cortical plate to lingual cortical plate distance.	Md1: 2 mm apical to L6 CEJ Md2: 6 mm apical to L6 CEJ Md3: 10 mm apical to L6 CEJ	Blue Sky Plan—Coronal sections
Symphysis width	Measured as described by Foosiri P et al. (2018) [13]. Distance from the most anterior point of the mandibular symphysis to the most convex lingual point.	Single measurement	Blue Sky Plan—Sagittal sections

CEJ, cementoenamel junction.

**Table 2:** Comparison of linear measurements obtained from integrated CBCT and conventional CBCT images.

	Variable	Integrated CBCT Mean (SD)	CBCT Mean (SD)	MD	95% CI	*p*-Value
Crestal level	UR1D1	7.626 (0.754)	7.611 (0.702)	0.016	−0.081–0.112	0.746
	UR4D1	9.800 (1.230)	9.615 (1.095)	0.185	0.080–0.290	0.001
	UR6D1	13.969 (1.887)	13.536 (1.705)	0.433	0.310–0.556	<0.001
	UL1D1	7.606 (0.866)	7.623 (0.702)	−0.017	−0.132–0.098	0.772
	UL4D1	9.812 (1.177)	9.540 (1.022)	0.272	0.165–0.379	<0.001
	UL6D1	13.896 (1.551)	13.468 (1.403)	0.428	0.318–0.538	<0.001
	LR1D1	6.046 (0.729)	6.186 (0.581)	−0.139	−0.246–−0.032	0.012
	LR4D1	8.112 (1.163)	8.095 (0.943)	0.017	−0.113–0.147	0.789
	LR6D1	11.253 (1.507)	11.017 (1.283)	0.236	0.078–0.394	0.004
	LL1D1	6.049 (0.690)	6.097 (0.612)	−0.049	−0.126–0.029	0.213
	LL4D1	8.237 (1.352)	8.154 (1.237)	0.083	−0.011–0.176	0.081
	LL6D1	11.381 (1.551)	10.987 (1.266)	0.394	0.217–0.571	<0.001
Mid-root level	UR1D2	8.246 (0.974)	8.012 (0.948)	0.234	0.165–0.303	<0.001
	UR4D2	9.550 (1.041)	9.262 (0.991)	0.288	0.203–0.372	<0.001
	UR6D2	14.386 (1.193)	13.928 (1.101)	0.458	0.357–0.559	<0.001
	UL1D2	8.180 (1.261)	7.923 (1.178)	0.258	0.147–0.368	<0.001
	UL4D2	9.610 (1.290)	9.161 (1.200)	0.449	0.347–0.552	<0.001
	UL6D2	14.359 (1.365)	13.896 (1.236)	0.464	0.367–0.560	<0.001
	LR1D2	6.012 (0.819)	5.926 (0.758)	0.086	0.020–0.152	0.012
	LR4D2	9.488 (1.716)	9.266 (1.695)	0.222	0.162–0.283	<0.001
	LR6D2	12.320 (1.720)	11.970 (1.684)	0.35	0.284–0.415	<0.001
	LL1D2	5.952 (0.795)	5.863 (0.710)	0.089	0.014–0.163	0.02
	LL4D2	9.577 (1.853)	9.281 (1.811)	0.296	0.225–0.366	<0.001
	LL6D2	12.362 (1.731)	12.004 (1.749)	0.357	0.200–0.514	<0.001
Apical level	UR1D3	9.202 (2.084)	8.773 (1.955)	0.428	0.212–0.645	<0.001
	UR4D3	9.378 (1.293)	8.920 (1.242)	0.458	0.391–0.524	<0.001
	UR6D3	15.457 (1.805)	14.845 (1.704)	0.612	0.508–0.715	<0.001
	UL1D3	9.424 (2.056)	8.873 (1.924)	0.552	0.388–0.716	<0.001
	UL4D3	9.247 (1.574)	8.661 (1.531)	0.586	0.499–0.673	<0.001
	UL6D3	15.308 (1.927)	14.745 (1.855)	0.563	0.447–0.678	<0.001
	LR1D3	7.410 (1.561)	7.091 (1.493)	0.318	0.264–0.373	<0.001
	LR4D3	9.794 (1.834)	9.476 (1.792)	0.317	0.260–0.375	<0.001
	LR6D3	12.298 (1.971)	11.992 (1.986)	0.305	0.247–0.363	<0.001
	LL1D3	7.316 (1.490)	7.152 (1.397)	0.208	0.145–0.271	<0.001
	LL4D3	9.811 (1.769)	9.472 (1.709)	0.339	0.271–0.407	<0.001
	LL6D3	12.150 (1.779)	11.826 (1.797)	0.324	0.234–0.414	<0.001
Root length	UR1L	21.540 (1.572)	22.796 (1.667)	−1.256	−1.428–−1.083	<0.001
	UR4L	16.465 (2.290)	17.767 (2.384)	−1.303	−1.468–−1.138	<0.001
	UR6L	18.400 (1.603)	19.411 (1.599)	−1.011	−1.143–−0.880	<0.001
	UL1L	22.014 (1.682)	23.142 (1.779)	−1.129	−1.258–−0.999	<0.001
	UL4L	16.741 (2.321)	17.800 (2.447)	−1.058	−1.183–−0.934	<0.001
	UL6L	18.447 (1.524)	19.628 (1.532)	−1.181	−1.363–−0.999	<0.001
	LR1L	19.136 (1.193)	20.733 (1.189)	−1.598	−1.843–−1.352	<0.001
	LR4L	18.765 (2.731)	20.132 (2.619)	−1.368	−1.606–−1.129	<0.001
	LR6L	17.463 (1.673)	18.668 (1.643)	−1.206	−1.348–−1.063	<0.001
	LL1L	18.701 (1.426)	20.379 (1.284)	−1.678	−1.975–−1.380	<0.001
	LL4L	18.432 (2.787)	19.702 (2.963)	−1.271	−1.568–−0.973	<0.001
	LL6L	17.770 (1.868)	18.808 (1.817)	−1.038	−1.155–−0.921	<0.001
Maxillary width	MXI1	30.002 (2.827)	30.383 (2.826)	−0.381	−0.491–−0.272	<0.001
	MXI2	28.029 (2.951)	28.501 (2.899)	−0.472	−0.561–−0.383	<0.001
	MXI3	25.364 (3.604)	25.965 (3.547)	−0.601	−0.714–−0.489	<0.001
	MXO1	56.583 (3.854)	56.055 (3.727)	0.529	0.381–0.677	<0.001
	MXO2	55.527 (4.421)	55.058 (4.396)	0.468	0.340–0.597	<0.001
	MXO3	55.346 (5.198)	54.706 (5.105)	0.641	0.495–0.786	<0.001
Mandibular width	MDI1	31.317 (3.481)	31.657 (3.453)	−0.34	−0.456–−0.224	<0.001
	MDI2	30.166 (4.034)	30.509 (3.993)	−0.342	−0.462–−0.222	<0.001
	MDI3	31.065 (4.582)	31.083 (4.679)	−0.018	−0.494–0.458	0.94
	MDO1	54.967 (3.475)	54.554 (3.455)	0.413	0.279–0.547	<0.001
	MDO2	58.015 (4.921)	57.382 (4.787)	0.632	0.494–0.771	<0.001
	MDO3	59.894 (5.893)	59.564 (5.922)	0.33	0.088–0.571	0.008
Symphysis	SM	15.232 (1.924)	15.101 (1.807)	0.131	0.046–0.216	0.003

MD, mean difference; SD, standard deviation; CI, confidence interval; CBCT, cone-beam computed tomography.

## Data Availability

The original contributions presented in this study are included in the article/Supplementary Materials. Further inquiries can be directed to the corresponding author.

## Supplementary Materials

The following supporting information can be downloaded at https://www.mdpi.com/article/10.3390/dj14090580/s1. Figure S1: Bland–Altman plots for crestal alveolar bone width measurements; Figure S2: Bland–Altman plots for mid-root alveolar bone width measurements; Figure S3: Bland–Altman plots for apical alveolar bone width measurements; Figure S4: Bland–Altman plots for root length measurements; Figure S5: Bland–Altman plots for maxillary transverse width measurements; Figure S6: Bland –Altman plots for mandibular transverse width measurements; Figure S7: Bland–Altman plot for symphyseal width measurements; Table S1: Numerical Bland–Altman bias and 95% limits of agreement for integrated CBCT versus conventional CBCT measurements.

## Abbreviations

The following abbreviations are used in this manuscript:

CBCT	cone-beam computed tomography
STL	standard tessellation language
