# Supplementary material for: Quantitative Comparison of Bone and Dental Morphology Between Invisalign® Integrated CBCT Images and Conventional CBCT Images
Source: Dent J (Basel). 2026 Sep 9;14(9):580. doi: 10.3390/dj14090580 (PMC13605112; doi:10.3390/dj14090580)
Supplement: Supplementary file 1 [file dentistry-14-00580-s001.zip › dentistry-4463840-supplementary.pdf]

Supplementary Figure S1 Bland–Altman plots for crestal alveolar bone width measurements

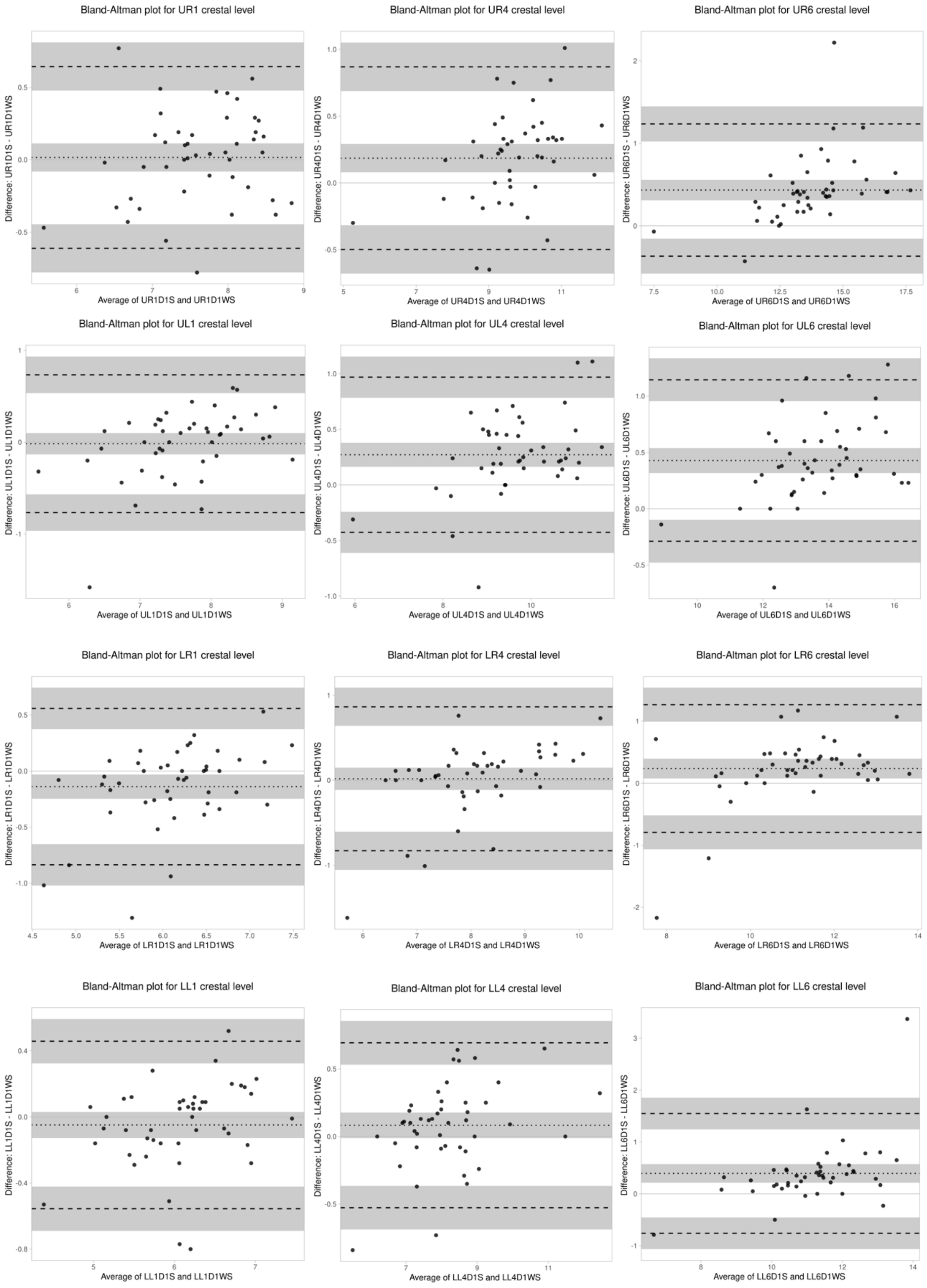

Supplementary Figure S2 Bland–Altman plots for mid-root alveolar bone width measurements

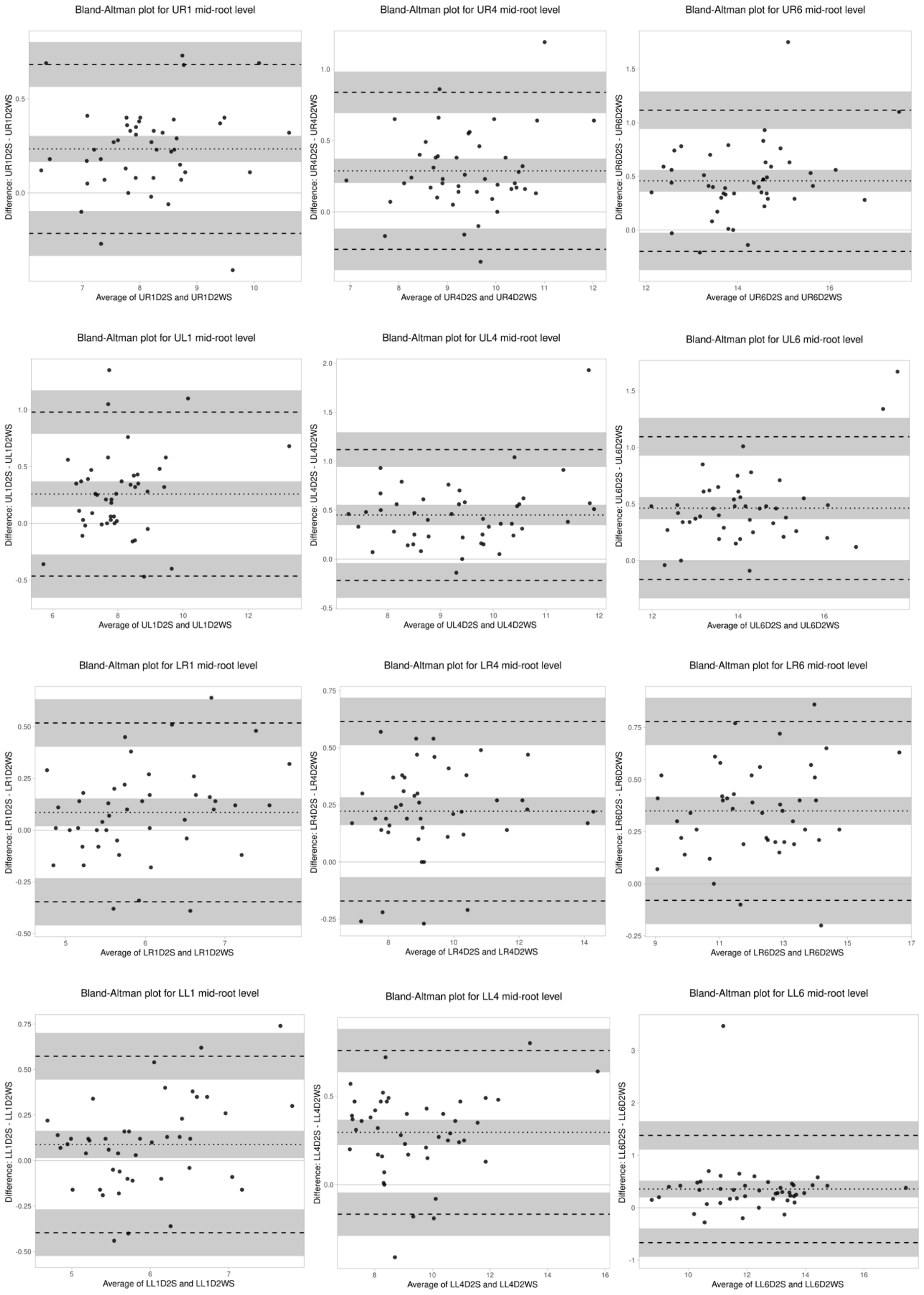

Supplementary Figure S3 Bland–Altman plots for apical alveolar bone width measurements

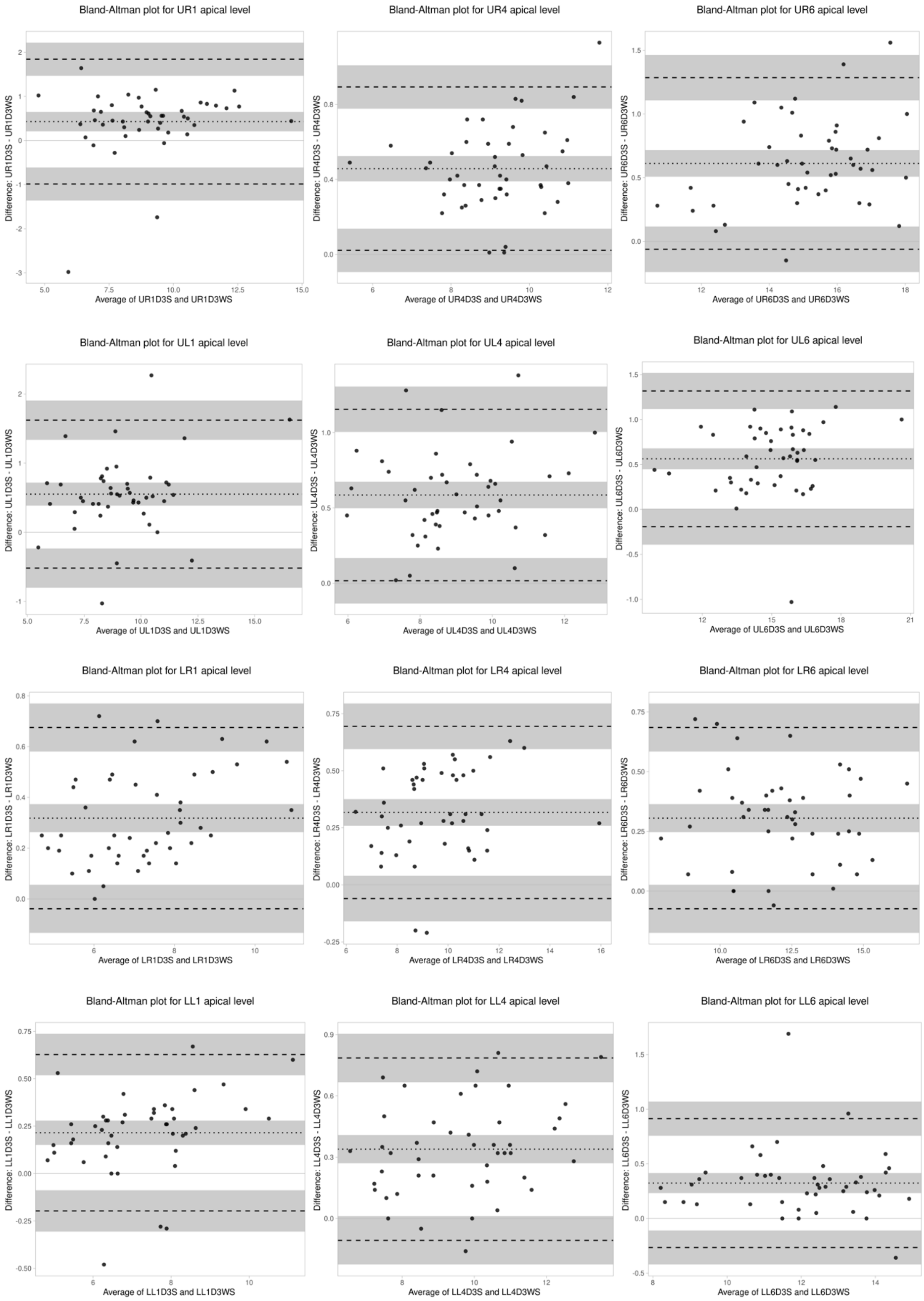

Supplementary Figure S4 Bland–Altman plots for root length measurements

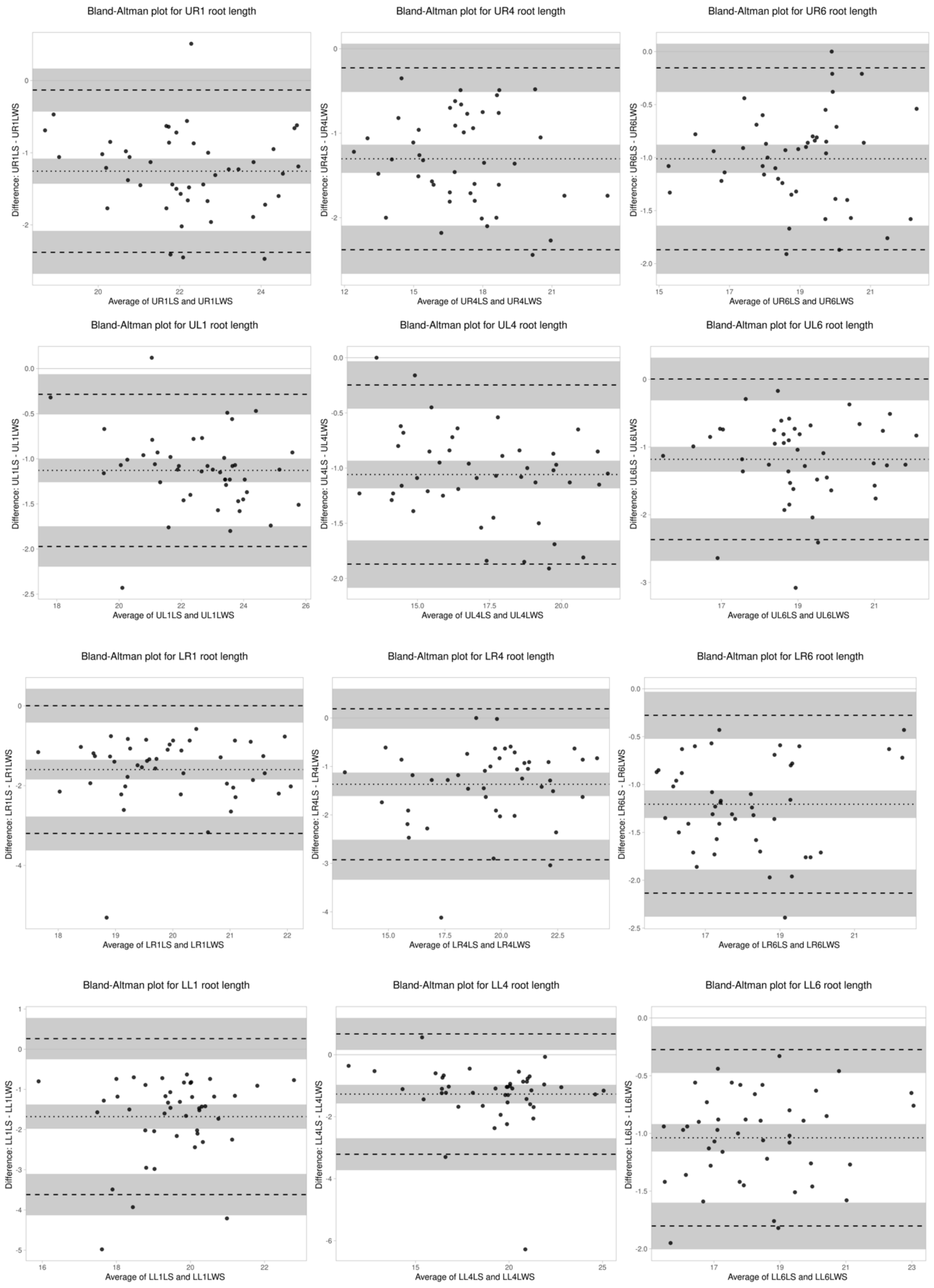

Supplementary Figure S5 Bland–Altman plots for maxillary transverse width measurements

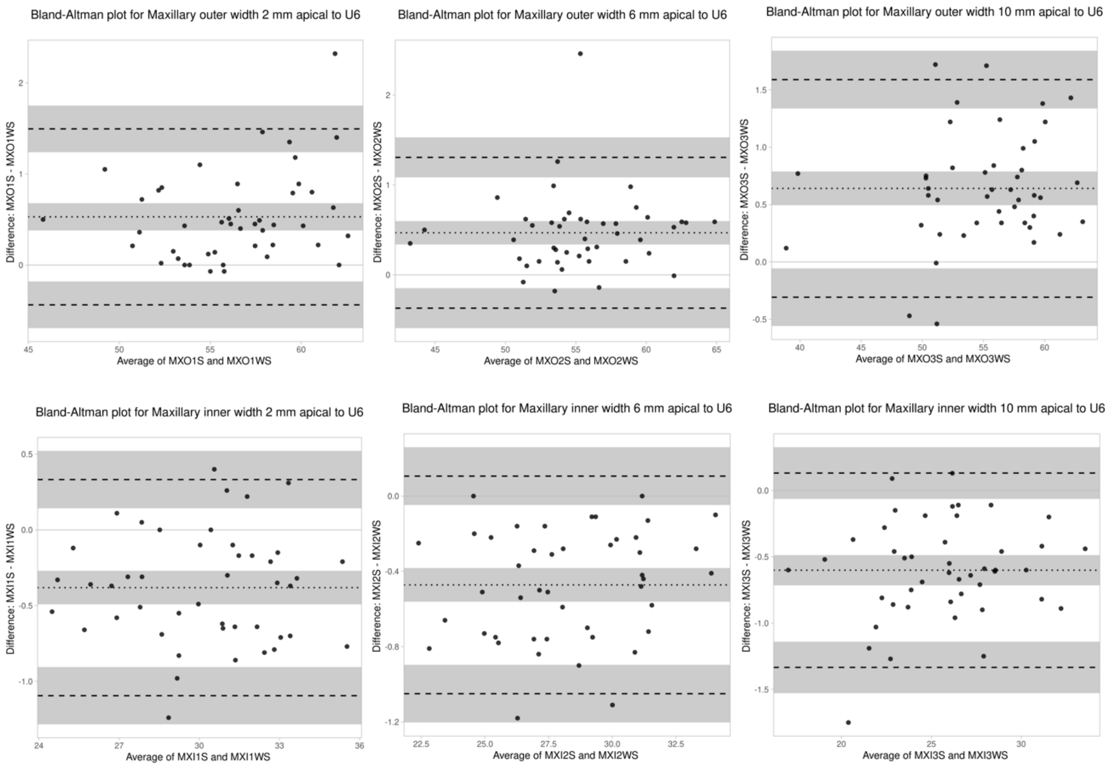

Supplementary Figure S6 Bland –Altman plots for mandibular transverse width measurements

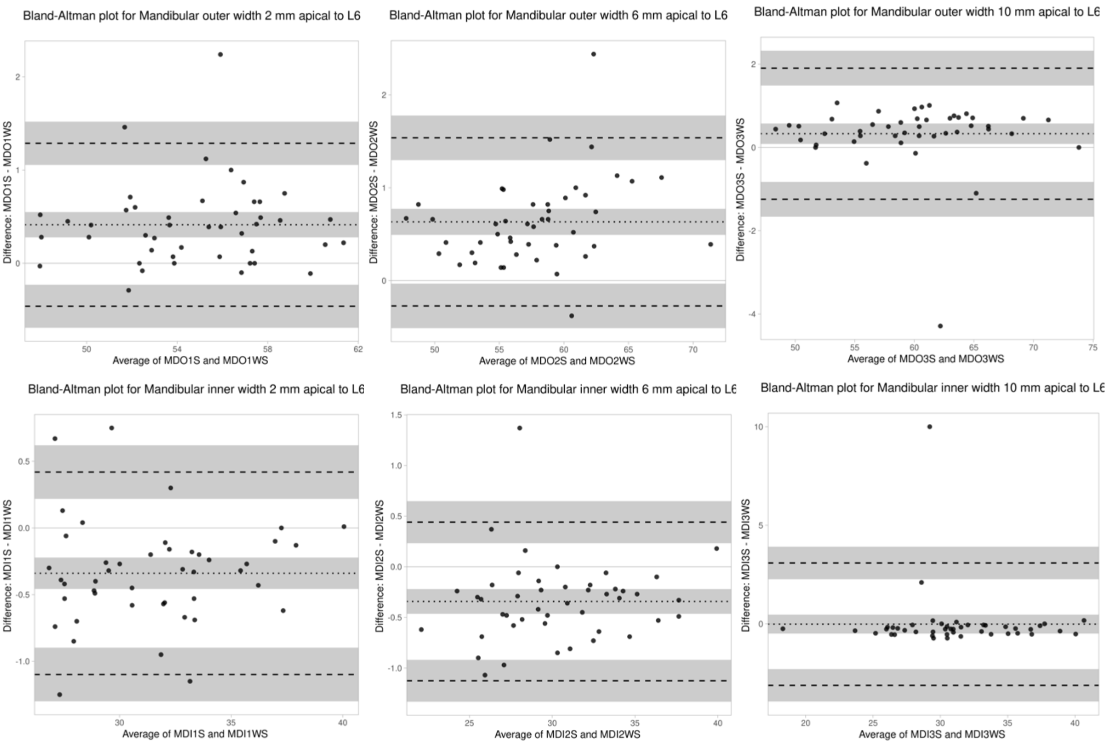

Supplementary Figure S7 Bland–Altman plot for symphyseal width measurements

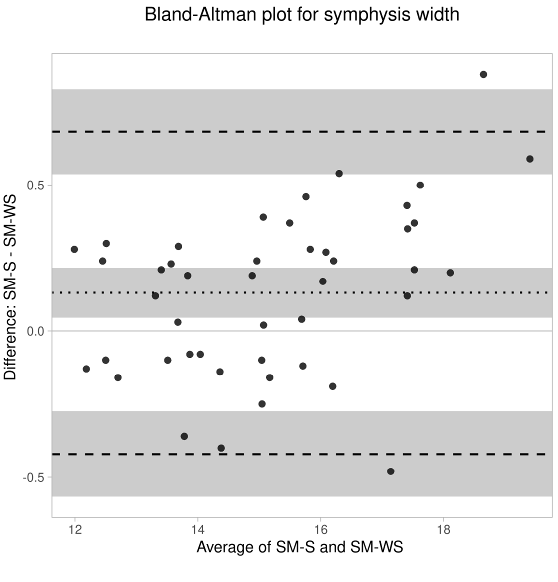

Supplementary Table S1. Numerical Bland–Altman bias and 95% limits of agreement for integrated CBCT versus conventional CBCT measurements.

Measurement group	Variable	Bias (mm)	Lower 95% LoA (mm)	Upper 95% LoA (mm)
Crestal level	UR1D1	0.016	-0.61	0.65
Crestal level	UR4D1	0.185	-0.50	0.87
Crestal level	UR6D1	0.433	-0.37	1.24
Crestal level	UL1D1	-0.017	-0.77	0.73
Crestal level	UL4D1	0.272	-0.43	0.97
Crestal level	UL6D1	0.428	-0.29	1.15
Crestal level	LR1D1	-0.139	-0.84	0.56
Crestal level	LR4D1	0.017	-0.83	0.87
Crestal level	LR6D1	0.236	-0.79	1.27
Crestal level	LL1D1	-0.049	-0.55	0.46
Crestal level	LL4D1	0.083	-0.53	0.69
Crestal level	LL6D1	0.394	-0.76	1.55
Mid-root level	UR1D2	0.234	-0.22	0.68
Mid-root level	UR4D2	0.288	-0.26	0.84
Mid-root level	UR6D2	0.458	-0.20	1.12
Mid-root level	UL1D2	0.258	-0.46	0.98
Mid-root level	UL4D2	0.449	-0.22	1.12
Mid-root level	UL6D2	0.464	-0.17	1.09
Mid-root level	LR1D2	0.086	-0.34	0.52
Mid-root level	LR4D2	0.222	-0.17	0.62
Mid-root level	LR6D2	0.350	-0.08	0.78
Mid-root level	LL1D2	0.089	-0.40	0.58
Mid-root level	LL4D2	0.296	-0.16	0.76
Mid-root level	LL6D2	0.357	-0.67	1.38
Apical level	UR1D3	0.428	-0.98	1.84
Apical level	UR4D3	0.458	0.02	0.89
Apical level	UR6D3	0.612	-0.06	1.29
Apical level	UL1D3	0.552	-0.52	1.62
Apical level	UL4D3	0.586	0.02	1.15
Apical level	UL6D3	0.563	-0.19	1.32
Apical level	LR1D3	0.318	-0.04	0.67
Apical level	LR4D3	0.317	-0.06	0.69
Apical level	LR6D3	0.305	-0.07	0.68
Apical level	LL1D3	0.208	-0.20	0.62
Apical level	LL4D3	0.339	-0.10	0.78
Apical level	LL6D3	0.324	-0.26	0.91
Root length	UR1L	-1.256	-2.38	-0.13

Root length	UR4L	-1.303	-2.38	-0.23
Root length	UR6L	-1.011	-1.87	-0.15
Root length	UL1L	-1.129	-1.97	-0.28
Root length	UL4L	-1.058	-1.87	-0.25
Root length	UL6L	-1.181	-2.37	0.01
Root length	LR1L	-1.598	-3.20	0.00
Root length	LR4L	-1.368	-2.92	0.19
Root length	LR6L	-1.206	-2.14	-0.28
Root length	LL1L	-1.678	-3.62	0.26
Root length	LL4L	-1.271	-3.21	0.67
Root length	LL6L	-1.038	-1.80	-0.27
Maxillary width	MXI1	-0.381	-1.10	0.33
Maxillary width	MXI2	-0.472	-1.05	0.11
Maxillary width	MXI3	-0.601	-1.33	0.13
Maxillary width	MXO1	0.529	-0.44	1.49
Maxillary width	MXO2	0.468	-0.37	1.31
Maxillary width	MXO3	0.641	-0.31	1.59
Mandibular width	MDI1	-0.340	-1.10	0.42
Mandibular width	MDI2	-0.342	-1.12	0.44
Mandibular width	MDI3	-0.018	-3.12	3.09
Mandibular width	MDO1	0.413	-0.46	1.29
Mandibular width	MDO2	0.632	-0.27	1.54
Mandibular width	MDO3	0.330	-1.25	1.91
Symphysis	SM	0.131	-0.42	0.69

LoA, limits of agreement; CBCT, cone-beam computed tomography.
